# Development of MY-DRG casemix pharmacy service weights in UKM Medical Centre in Malaysia

**DOI:** 10.1186/s40199-014-0075-4

**Published:** 2015-02-10

**Authors:** Saad Ahmed Ali Jadoo, Syed Mohamed Aljunid, Amrizal Muhammad Nur, Zafar Ahmed, Dexter Van Dort

**Affiliations:** United Nations University-International Institute for Global Health (UNU-IIGH), Kuala Lumpur, Malaysia; International Centre for Case-Mix and Clinical Coding (ITCC), University Kebangsaan Malaysia Medical Centre, Jalan Yaacob Latiff, 56000 Cheras, Kuala Lumpur, Malaysia; Pharmacy of Hospital University Kebangsaan Malaysia Medical Centre, Jalan Yaacob Latiff, 56000 Cheras, Kuala Lumpur, Malaysia

**Keywords:** Diagnosis related groups, Pharmacy, Service weight, Malaysia

## Abstract

**Background:**

The service weight is among several issues and challenges in the implementation of case-mix in developing countries, including Malaysia. The aim of this study is to develop the Malaysian Diagnosis Related Group (MY-DRG) case-mix pharmacy service weight in University Kebangsaan Malaysia-Medical Center (UKMMC) by identifying the actual cost of pharmacy services by MY-DRG groups in the hospital.

**Methods:**

All patients admitted to UKMMC in 2011 were recruited in this study. Combination of Step-down and Bottom-up costing methodology has been used in this study. The drug and supplies cost; the cost of staff; the overhead cost; and the equipment cost make up the four components of pharmacy. Direct costing approach has been employed to calculate Drugs and supplies cost from electronic-prescription system; and the inpatient pharmacy staff cost, while the overhead cost and the pharmacy equipments cost have been calculated indirectly from MY-DRG data base. The total pharmacy cost was obtained by summing the four pharmacy components’ cost per each MY-DRG. The Pharmacy service weight of a MY-DRG was estimated by dividing the average pharmacy cost of the investigated MY-DRG on the average of a specified MY-DRG (which usually the average pharmacy cost of all MY-DRGs).

**Results:**

Drugs and supplies were the main component (86.0%) of pharmacy cost compared o overhead cost centers (7.3%), staff cost (6.5%) and pharmacy equipments (0.2%) respectively. Out of 789 inpatient MY-DRGs case-mix groups, 450 (57.0%) groups were utilized by the UKMMC. Pharmacy service weight has been calculated for each of these 450 MY-DRGs groups. MY-DRG case-mix group of Lymphoma & Chronic Leukemia group with severity level three (C-4-11-III) has the highest pharmacy service weight of 11.8 equivalents to average pharmacy cost of RM 5383.90. While the MY-DRG case-mix group for Circumcision with severity level one (V-1-15-I) has the lowest pharmacy service weight of 0.04 equivalents to average pharmacy cost of RM 17.83.

**Conclusion:**

A mixed approach which is based partly on top-down and partly on bottom up costing methodology has been recruited to develop MY-DRG case-mix pharmacy service weight for 450 groups utilized by the UKMMC in 2011.

## Background

Drug costs constitute the majority of health system pharmacy budgets and continue to increase faster than other health care expenditures [1]. It is accounting for more than 15.2% of total health expenditure in the world in 2000 [2] and almost a fifth of all health spending on average across OECD countries [3]. In 2006, Pharmaceutical spending ranges from a mean of (19.7% to 30.4%) in the high-income countries and the low-income countries respectively as a share of total health expenditure [4]. However, one third of the world population lacks reliable access to essential drugs [2].

Falkenberg and Tomson [5] indicated that “around 50% of all medicines worldwide are prescribed, dispensed, or sold inappropriately”. These inefficient and ineffective uses of medicines make it continuously a target for cost control, management evaluation and policy regulation [1,6].

The development of Diagnosis Related group (DRG) in 1960s as a system comparing resource utilization across groups of patients with the same principal diagnosis greatly facilitated pharmacoeconomic evaluation [7]. The major determinant of pharmacy expenditure in any health institution is the patient complexity, so for a more effective drug costs control, methods for case mix adjustment should be considered [8].

Today more than 40 countries worldwide implemented case-mix system for various purposes and in varying levels [9,10]. The importance of the Case mix and associated cost weights is directly proportional with the increasing demand for the development of new hospital funding methodologies in many countries. The integrity of both the case-mix grouper algorithm employed and the associated relative cost weights has a direct impact on the integrity of these new funding methodologies. While, the calculation of cost weights and the development of a case-mix grouper depends on the availability of patient level case cost [11,12].

Cost is the resources spent to generate the benefits. Resources may be in the form of money, time, labour or other resources used to produce a product such as health services. However, the calculation of actual costs is not easy issue and commonly it is based on best estimates and averages across the hospital system. It was noted that during the implementation of case-mix based payment systems the attention was focused on the coding and generation of accurate and comprehensive DRG activity data. This interest is mainly because the methodologies analyzing activity patterns are well established and standards for DRG classification and coding are well documented, as well as the accurate patient’s records that have been properly coded into grouper make the case-mix data quite acceptable for the purpose of defining hospital production. Thus, the costing of hospital services including the pharmacy is often neglected unintentionally. The price is different to cost, but without the understanding of costs, pricing is not possible. If prices are difficult to set, then payment models that fairly pay for what hospitals produce cannot be formulated [13,14].

UKMMC is one of the leading hospitals in Malaysia that implemented the case-mix system in 2002, as an appropriated provider payment mechanism, in line with continues national health reform process towards the provision of equitable and efficient health services [15]. “Cost weights or the relative weights are an important component of prospective payment system, since they provide the variation in payment levels that reflect the relative resources required for visits within each classification group” [16]. Cost weight was among several issues and challenges that faced the implementation of case-mix in Malaysia [17]. This study aimed to develop the pharmacy service weights in UKMMC by identifying the pharmacy services and the actual cost of care.

## Methodology

### Study background

This was a retrospective study with data collected from inpatients pharmacy electronic prescription and the Case-Mix database of University Kebangsaan Malaysia Medical Center (UKMMC) of year 2011. UKMMC was formed as a result of the amalgamation of the Faculty of Medicine and Hospital of University Kebangsaan Malaysia (HUKM) in early 2008. The Centre provides a broad range of teaching and tertiary referral services in over 1050 licensed inpatient beds, supported by extensive outpatient services, in addition to the primary emergency reception centre for the south eastern suburbs of Kuala Lumpur, the capital city of Malaysia. In 2011, there were 384, 496 outpatient and 35, 303 inpatient occasions of services reported in this hospital (i.e. number of admissions or number of admitted patients). The total budget of the UKMMC was in excess of 473 million Malaysian Ringgit (USD 150 million) with a 3453 total number of staff and covered area of 90203.00 (m2). Currently the UKMMC consists of a hospital, Faculty of Medicine, Institute of Medical Molecular Research (UMBI) and affiliated with UKM for teaching undergraduate and post graduate medical students [18,19].

### Profile of UKMMC pharmacy

The UKMMC pharmacy is in charge of pharmacy services for all in and outpatients of UKMMC. The total pharmacy budget (drugs and supplies only) of 2011 was in excess of RM 89,870,771 equivalent to USD 28,466,240 (Exchange Rate of 27^th^ August 2014) with a 123 total number of staff and covering area of 2187 (m2). The UKMMC pharmacy office allocated almost 16% (RM 13, 880, 484.98) of the annual pharmacy drugs and supplies budget to inpatient services and recruiting only 16% (20 persons) of the total staff to manage and distribute inpatient services [19].

### Sample size and study period

The study used information on the pharmacy costs of all patients admitted to UKMMC from 1^st^ January to 31^st^ December 2011, comprising the 35, 303 separations. The average length of stay (ALOS) of these patients was 5.5 days, representing 193,824 days of patient care [19].

### DRG assignment

In this study, over 20,192 inpatient electronic-prescriptions with ALOS of 6.6 days were assigned a DRG. There were 633 DRGs identified in the study, 10.3% of which had only 1 case and 28.9% had less than 5 cases. DRG O-6-12-I, Vaginal Delivery with other Procedure Excluding Sterilization &/Or Dilation & Curettage (3.75%), was the highest volume DRG identified in this study. The range of length of stay varied from 1 to 69 days, and the highest proportion of patients (16.4%) and (20.6%) separated on the second and third day after admission respectively.

### Data issues

The following steps have been done to develop the MY-DRG case-mix pharmacy service weight in UKMMC by identifying the actual cost of pharmacy services by MY-DRG groups in the hospital.

### Step one: identifying the pharmacy component

In order to estimate the total costs of a particular health service, it is important to identify all the relevant costs and those who bear these costs [20]. In this study the pharmacy component has been identified to include four main contributors:

- The drugs and supplies cost.
- The cost of in-patient pharmacy staff.
- The overhead cost centres allocation.
- The pharmacy equipment cost.

### Step two: calculation of the total pharmacy cost

A mixed approach which is based partly on top-down (step-down) and partly on bottom up or activity based (ABC) costing methodology has been recruited in order to calculate the pharmacy cost per patient or episodes [15,21]. The required data (retrospective data) for ABC approach were the all inpatient e-prescriptions and the total number of the inpatient pharmacy staff for year 2011 in UKMMC. While the data needed for top down costing were the total of hospital expenditures, total number of hospital staff, total hospital floor area and total number of inpatients for year 2011, in UKMMC.

- Top down costing:
  The main purpose of this step is to determine the pharmacy use of the indirect (overhead) cost centers. Normally starts (at the top) with total expenditures and then divides these by a measure of total output (e.g. patient visits, days or admissions) to give an “average” cost per patient per visit, per day or per admission [22,23]. The top-down approach is cheaper and faster than a bottom up approach because of less data intensive and fewer research skills needed and data can be collected from routine resources [15].

#### The pharmacy use of the overhead cost centers and allocation factors

**Figure 1:**
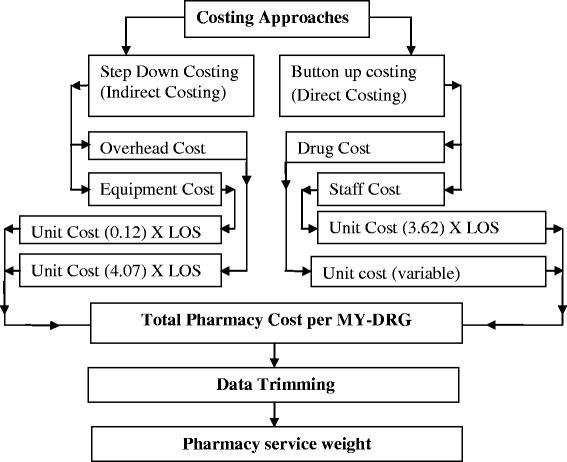
**Study costing strategy.**

The overhead cost centers included in this study are the administration, maintenance, utility, cleaning services, security, general store and consumable, information technology (IT) centre, library, tax and insurance, rent, The central sterile services department (CSSD), telephone and fax centers. Data on the annual total cost for each center has been collected from the financial department of UKMMC. Table 1 shows the overhead cost centers and the appropriate allocation factors used to determine the pharmacy use of the indirect (overhead) cost centers. The following questions have been used to calculate the pharmacy use of each of the overhead cost centers: The pharmacy use of the indirect (overhead) cost centers = (Number of pharmacy staff/total hospital staff) × (annual total cost) or the pharmacy use of the indirect (overhead) cost centers = (pharmacy floor area/total hospital floor area) × (annual total cost). Summing up all the allocations gave the total pharmacy use of the indirect (overhead) cost centers. Then the total pharmacy use of the indirect (overhead) cost centers multiplied by the inpatient proportion to get the inpatient pharmacy use of the indirect (overhead) cost centers. The results of this question divided by total annual number of (inpatient days) to get the inpatient pharmacy use cost per day. This unit cost in the question was then multiplied by (the length of stays) of investigated patient to get the inpatient pharmacy use cost per patient per day, Figure 1.

**Table 1:** **Overhead cost centers and the allocation key factors**

**No.**	**Allocation factor**	
	**Number of staff**	**Floor area**
1	The administrative,	The maintenance
2	General store and consumable	Utility
3	IT centre	Cleaning Services
4	Library	Security
5	Tax and insurance	CSSD centers
6	Rent	
7	Telephone and fax centers

#### The pharmacy equipment cost (capital costs)

In this costing study the pharmacy capital costs comprised of all the purchased or donated equipments, furniture and vehicles in the last 5 years (RM 642,375.16). The information was obtained from the financial department in the hospital. The total capital cost has been divided by Annualization factor (4.32) at 5% discount rate [24]. The result (RM148, 697.95) has been multiplied by inpatient proportion (16%) which already determined by The UKMMC pharmacy office. Then the inpatient pharmacy capital asset costs (RM 23, 791.67) divided by the total number of inpatient days (193824) to get the capital assets cost per day. This unit cost (0.12) in the question was multiplied by length of stays of the investigated patient to get the inpatient pharmacy capital assets cost per patient per day.

b. Bottom-up or Activity based costing and data collection:
  The Bottom-up costing requires recording of every item of service that a patient receives, and changing them into costs. Bottom-up costing gives more accurate results, but it requires a large investment in time and resources [24,25]. In this study the bottom-up approach was used 1) to estimate drug and supply cost per episode and 2) to cost inpatient staff of the pharmacy per patient.

#### Costs of pharmacy staff serviced inpatients

Direct cost for staff cost covered all in-patient pharmacy staff responsible for supplying medications to all wards and units in UKMMC. Basic salaries and additional allowances, bonuses, contributions, payments were obtained from the staff directly and confirmed by hospital personnel services administrative records. Summing up all in-patient pharmacy staff costs gave the total staff cost (RM 702,030.48). Then the total staff cost was divided by total annual number of inpatient days (193824) to get staff cost per day. This unit cost (3.62) was then multiplied by (length of stay) of investigated patient to estimate staff cost per patient per day.

#### Cost of inpatient drugs/medicines and medical supplies

Information on the drugs/medicines, fluids and medical supplies prescribed to the patient was obtained from electronic-prescription system on Excel based file. Electronic prescribing refers to the ordering, administration and supply of drugs is completely supported by electronic systems. Each e-prescription defined as one episode (a period of inpatient care) [26] having data on registration number (MRN) which is a unique number given for each local and international client; name of the patient; e-prescription number; date of prescription; number of items, quantity, duration and name of the prescribed medicines, fluids and supplies. Drugs prescribed and purchased by patients for take home were excluded. List of acquisition unit costs (price) of each drug and supplies were obtained from the UKMMC pharmacy office. This unit cost was then multiplied by the quantity of the corresponding item to estimate cost per item. Then we summed up the cost of all items in one e-prescription to estimate drug cost per patient (per episode).

#### Calculation of patient level total pharmacy costs

The total pharmacy cost of each individual patient/episode would be the summing of total drugs and supplies cost plus the results of multiplying the unit cost of each of pharmacy use of overhead cost centers; the pharmacy equipment cost; pharmacy staff cost by the LOS of investigated patient/episode.

### Step three: data trimming

The L3H3 method (Lower three Higher three), is data trimming method commonly used to ensure that the means reported more accurately represent the central tendency amongst cases analyzed [27]. For each DRG we calculated the total and the average pharmacy cost depending on the number of patients/episodes in that DRG. Trimming method mainly consists of using the average pharmacy cost for every MY-DRG having more than 20 patients/episodes divided by three as the low trim point and the average pharmacy cost multiplied by three as the high trim point. So, in term of distribution the normal cases in each MY-DRG lie inside the trim points and known as inliers. In contrast, the cases which lie outside the trim points considered skewed or outlier cases and have been excluded from analysis.

### Step four: calculation the pharmacy service weight per each MY-DRG

Pharmacy service weight was defined as the burden of work or services performed by pharmacy component and the resources used for a patient compared to the burden of other services for others DRGs. The actual service weights are unit less numbers that express the expected cost for one visit in relation to average visit. The best way to calculate service weights is to use actual cost per inpatient case by assigning each DRG a relative value that reflects the cost of any one, or all, of the resources consumed (e.g. bed-days, theatre time, drugs, diagnostic procedures, physiotherapy and nursing treatment) in that respective DRG when compared with all DRGs [28,29]. In order to estimate the pharmacy service weight we need first to calculate the average pharmacy cost for all MY-DRGs. The closest average cost among all the MY-DRGs would be the base used to calculate the pharmacy service weight using the following question:

Pharmacy service weight of a MY-DRG = Average pharmacy cost of the investigated MY-DRG/Average of a specified MY-DRG (which usually the average pharmacy cost of all MY-DRGs).

### Ethics

This study was approved by ethics committee of National University of Malaysia- Medical Center (UKMMC), code number (UNU-002- 2013) in 20 May 2013.

## Results

### The pharmacy component unit costs

Table 2 shows the estimated unit costs of the pharmacy components with exception of the drugs and supplies unit cost which is variable depending on the number of items and the quantity of drugs and supplies consumed by each patient (episode).

**Table 2:** **Unit costs of the four pharmacy components**

**No.**	**Pharmacy components**	**Unit cost (RM)**
1	Total drugs and supplies cost	Episode/variable
2	Pharmacy staff cost	3.62
3	Pharmacy use of overhead cost centers	4.07
4	Pharmacy equipment cost	0.12

### The pharmacy component

The total and the average pharmacy cost have been calculated for each MY-DRG using the estimated unit costs. Table 3 reports the frequency distribution of the average pharmacy components cost: Drugs and supplies were the main component (86.0%) of pharmacy cost compared to overhead cost centers (7.31%), staff cost (6.50%) and pharmacy equipments (0.22%) respectively.

**Table 3:** **Frequency distribution of the average pharmacy components cost**

**No.**	**Pharmacy components**	**Average (RM)**	**%**
1	Total drugs and supplies cost	315.15	85.98
2	Pharmacy staff cost	23.81	6.50
3	Pharmacy use of overhead cost centers	26.78	7.31
4	Pharmacy equipment cost	0.79	0.22
	Average total pharmacy cost (n = 20,192)	366.53	100

### After data trimming

After data trimming and excluding DRGs with less than 5 cases, 13,663 cases with ALOS of 7 days were available for analysis. There were 450 DRGs identified in the study, 5.6% of which had only 5 cases. DRG O-6-13-I, Vaginal Delivery with severity level one (5.0%) was the highest volume DRG identified in this study. Almost 61.3% of total separations were classified as medical and 39.7% of them were classified into the surgical partition.

### Pharmacy service weight

Average pharmacy cost of all MY-DRGs was 484.48. MY-DRG F-4-16-III, Dementia and Other Organic Brain Disturbances Including Mental Retardation with severity level three was the closest average (486.08) among all other MY-DRGs. Thus this average was the base (denominator) used in the question to estimate the pharmacy service weight for all MY-DRGs. Table 4 shows the pharmacy service weight of the highest 20 MY-DRGs. MY-DRG case-mix group of Lymphoma & Chronic Leukemia group with severity level three (C-4-11-III) has the highest pharmacy service weight of 11.8 equivalents to average pharmacy cost of RM 5383.90. While the MY-DRG case-mix group for Circumcision with severity level one (V-1-15-I) has the lowest pharmacy service weight of 0.04 equivalents to average pharmacy Cost RM 17.83.

**Table 4:** **Pharmacy service weights of the highest 20 MY-DRGs**

**No.**	**MY-DRG**	**No. of episodes per DRG**	**Total pharmacy cost per DRG**	**Average pharmacy cost per DRG**	**Pharmacy service weight**
1	C-4-11-III	38	204588.16	5383.90	11.8
2	B-1-10-III	11	49817.84	4528.89	9.32
3	J-1-20-III	5	22178.34	4435.67	9.13
4	U-1-20-III	17	61664.92	3627.35	7.46
5	M-1-20-III	5	16848.74	3369.75	6.93
6	C-4-10-III	31	104232.86	3362.35	6.92
7	G-1-11-III	14	46170.40	3297.89	6.78
8	M-1-60-III	8	22978.67	2872.33	5.91
9	S-4-13-III	6	17148.36	2858.06	5.88
10	D-4-10-III	13	34282.24	2637.10	5.43
11	B-1-11-III	7	18080.84	2582.98	5.31
12	M-1-03-III	11	26994.90	2454.08	5.05
13	J-4-12-III	9	20444.37	2271.60	4.67
14	G-4-21-III	5	10925.50	2185.10	4.50
15	D-1-10-I	9	18569.22	2063.25	4.24
16	I-4-13-III	6	11693.31	1948.89	4.01
17	I-1-04-III	5	9550.77	1910.15	3.93
18	D-1-20-III	5	9455.59	1891.12	3.89
19	K-1-20-III	16	30134.09	1883.38	3.87
20	I-4-14-III	10	18375.21	1837.52	3.78

### Limitation of study

This study has few areas of limitations. First of all, this study was not designed to cover a representative size of hospitals in Malaysia due to time and resource constraints in addition to the limited number of hospitals that implemented DRG system in Malaysia. Other limitation is related to (date) of e-prescription issue which was not always be the same date of patient admission. This limitation made the joining of e-prescription data to MY-DRG data base to be done manually.

## Discussion

The main objective of this study was to develop the MY-DRG inpatient pharmacy service weight in UKMMC using E-prescription data and DRG data base in UKMMC. For this purpose a mixed approach of top-down and bottom-up costing methodology has been recruited jointly [24].

Although international literature indicated that there are several approaches to estimate the cost of providing services by health related institution including hospitals. However, there is no unique, appropriate and acceptable methodology for costing hospital services [30]. Type of the service and reason for costing in addition to economical feasibility of cost calculation are the main determinants for selection of appropriate costing approach. Thus, the cost of a particular service can vary substantially according to the purpose of cost data for which it was generated [31].

This study indicated that the drugs and supplies made the highest component of the pharmacy cost. These findings come in line with other international and local studies considering the pharmacy services as ancillary services [27] and among the highest components of cost in the hospital [28,29,32]. To our knowledge, the pharmacy services and its related weights are commonly studied within the general hospital level costing and are rarely to be evaluated as an independent subject [27,33].

Costing study done in Philippine for selected hospitals used both the activity based and top down costing approaches found that medicines and supplies cost more than 25% of the total hospital cost [33]. In 2004, two studies done in UKMMC, and the costing analysis were conducted based on the case-mix concept of the top-down costing approach. The first one was to study the cost analysis for cardiology. This study found that the three biggest components of medical cardiology cases are ICU cost (38.0%), Pharmacy component (14.2%) and Ward Services (12.7%). In the Surgical Cardiology, the biggest component of cost was the Operation Theatre (27.9%), followed by ward Services (25.4%) and Pharmacy Component (8.5%) [15]. The other study was the cost analysis and cost weight for the treatment of orthopedic cases in HUKM. This study showed that the top three components of cost for the treatment of medical orthopedic was Pharmacy Services (22.3%), followed by Ward Services which was (20.7%) and Laboratory Services which was (12.1%), while the top three components of cost for treatment of surgical orthopedic was Operating Theatre Services which was (21.2%), followed by Pharmacy Services which was (17.6%) and Ward Services which was (16.3). It is noted that for both the medical and surgical partitions of cardiology and orthopedic cases, the pharmacy component services were among the top three contributors of the large portion of cost or resources [34].

Indeed, the actual drug costs and the pharmacy cost are being among the main objectives of much research in the health care economy [29,35]. Although there is considerable variation between countries, the developing countries (the upper middle-income, the lower middle-income and the low-income countries) contribute only to 21.5% of the total global pharmaceutical expenditures in 2006; however they spend proportionally more of their health budget on medicines than the developed countries [4,36].

This study shows that using of e-prescription data would greatly facilitate the activity based costing methodology to estimate the pharmacy service weight by identifying pharmacy services and the actual cost of care. In fact, the availability of pharmacy service weights or cost weights will enable a comparison is made between the treatments cost of various DRG cases within and between hospitals [17]. For the purpose of using DRG as a base of hospital payment, a price needs to be assigned to each DRG. This is usually done by assigning a cost relativity (or service weight) with a base price multiplier [13].

## Conclusion

A mixed approach which is based partly on top-down and partly on bottom up costing methodology has been recruited to develop MY-DRG case-mix inpatient pharmacy service weight for 450 groups utilized by the UKMMC in 2011. This methodology can be used for calculating pharmacy service weight among Government Hospital such as General, District and private hospital in future. It is a hope that the results of this study will participate in the development of MY-DRG in UKMMC specifically in pharmacy services by identifying which DRG consumes the bulk of the resources. So, this can greatly support decision maker regarding budget planning of pharmacy services and patients’ outcomes, and eventually will contribute in the quality of care and services improvement as well as an effective use of resources.

## Abbreviations

MY-DRG: Malaysian Diagnosis Related Group
DRG: Diagnosis Related Group
UKMMC: University Kebangsaan Malaysia Medical center
HUKM: Hospital OF University Kebangsaan Malaysia
UMBI: Institute of Medical Molecular Research
ALOS: The average length of stay
L3H3: L three H three
IT: Information technology
CSSD: The Central Sterile Services Department
